# Increased Inhibition of the Amygdala by the mPFC may Reflect a Resilience Factor in Post-traumatic Stress Disorder: A Resting-State fMRI Granger Causality Analysis

**DOI:** 10.3389/fpsyt.2018.00516

**Published:** 2018-10-22

**Authors:** Feng Chen, Jun Ke, Rongfeng Qi, Qiang Xu, Yuan Zhong, Tao Liu, Jianjun Li, Li Zhang, Guangming Lu

**Affiliations:** ^1^Department of Radiology, Hainan General Hospital, Haikou, China; ^2^Department of Medical Imaging, Jinling Hospital, Medical School of Nanjing University, Nanjing, China; ^3^Department of Neurology, Hainan General Hospital, Haikou, China; ^4^Mental Health Institute, The Second Xiangya Hospital, National Technology Institute of Psychiatry, Key Laboratory of Psychiatry and Mental Health of Hunan Province, Central South University, Changsha, China

**Keywords:** post-traumatic stress disorder, amygdala, effective connectivity, medial prefrontal cortex, functional magnetic resonance imaging

## Abstract

**Purpose:** To determine whether effective connectivity of the amygdala is altered in traumatized subjects with and without post-traumatic stress disorder (PTSD).

**Materials and Methods:** Resting-state functional MRI data were obtained for 27 patients with typhoon-related PTSD, 33 trauma-exposed controls (TEC), and 30 healthy controls (HC). Effective connectivity of the bilateral amygdala was examined with Granger causality analysis and then compared between groups by conducting an analysis of variance.

**Results:** Compared to the HC group, both the PTSD group and the TEC group showed increased effective connectivity from the amygdala to the medial prefrontal cortex (mPFC). The TEC group showed increased effective connectivity from the mPFC to the amygdala relative to the HC group. Compared to the TEC group, the PTSD group showed increased effective connectivity from the amygdala to the supplementary motor area (SMA), whereas decreased effective connectivity was detected from the SMA to the amygdala. Both the PTSD group and the TEC group showed decreased effective connectivity from the superior temporal gyrus (STG) to the amygdala relative to the HC group. Compared to the HC group, the TEC group showed increased effective connectivity from the amygdala to the dorsolateral prefrontal cortex (dlPFC), while both the PTSD group and the TEC group showed decreased effective connectivity from the dlPFC to the amygdala. The PTSD group showed decreased effective connectivity from the precuneus to the amygdala relative to both control groups, but increased effective connectivity from the amygdala to the precuneus relative to the HC group.

**Conclusion:** Trauma leads to an increased down-top excitation from the amygdala to the mPFC and less regulation of the amygdala by the dlPFC. The results suggest that increased inhibition of the amygdala by the mPFC may reflect a resilience factor, and altered amygdala-SMA and amygdala-STG effective connectivity may reflect compensatory mechanisms of brain function. These data raise the possibility that insufficient inhibition of the amygdala by the mPFC might lead to PTSD in those who have been exposed to traumatic incidents, and may inform future therapeutic interventions.

## Introduction

After an exposure to traumatic events, most individuals will experience flashbacks, avoidance of trauma-related cues, sleep disorders, or other symptoms. Normally, these symptoms lessen or disappear within a few weeks, but some trauma-exposed individuals may experience a slow recovery and develop post-traumatic stress disorder (PTSD) (1, 2). The characteristics of PTSD mainly include intrusive thoughts (flashbacks or nightmares), attempts to avoid traumatic events, persistent hyper-vigilance, hypoemotivity, and mild cognitive decline (3). Apart from these, more than half of PTSD patients succumb to substance abuse, depression, and various anxiety disorders (4, 5), and more than a third progress into chronic, life-long, unhealed PTSD, which results in serious damage to social functioning.

Numerous neuroimaging studies have confirmed that functional and structural abnormalities are present in multiple brain areas of PTSD patients (6–8). Many PTSD fMRI (functional magnetic resonance imaging) studies, based on symptom provocation and cognitive activation, have found that, when compared with trauma-exposed subjects or healthy controls, PTSD patients showed increased activation in their amygdala and decreased activation in their medial prefrontal cortex (mPFC) (6, 7). Other brain areas that often show functional abnormalities include the insula, the dorsal anterior cingulate gyrus, the dorsolateral prefrontal cortex (dlPFC), the hippocampus, and the precuneus (6, 9). Also, structural MRI studies have found that the hippocampus, the parahippocampal gyrus, and the mPFC showed decreased gray matter volume, as well as reduced cortical thickness (10, 11). Based on these results and animal studies, many scholars have proposed various neural circuit models (12), of which the most widely accepted one is that PTSD patients may experience decreased inhibition of the amygdala by the mPFC, and hyper-responsiveness of the amygdala leads to an enhanced fear response. In addition, abnormal hippocampal function is associated with declarative memory impairment (13, 14).

Partially consistent with this neural circuit model, many PTSD fMRI studies, performed during the task- or resting-state, found decreased functional connectivity between the amygdala and the mPFC (7, 15, 16). Based on fMRI and emotion-processing tasks, Stevens et al. found that the PTSD group showed decreased functional connectivity between the amygdala and the mPFC, which indicates an abnormal regulation of the amygdala by the mPFC, compared to trauma-exposed controls (TECs) with no PTSD (17). Sripada et al. studied the brain function of PTSD in combat-exposed male patients in the resting-state. They found that, compared to the TECs with no PTSD, the negative functional connectivity between the amygdala and the mPFC was decreased, and there was an increased positive functional connectivity between the amygdala and the insula. However, a reduction in the positive connectivity between the amygdala and the hippocampus was observed in PTSD patients (15). In our other unpublished study, we also found a decreased negative functional connectivity between the amygdala and the mPFC among PTSD patients, but, due to the limitations of functional connectivity analysis, none of the above studies could ascertain the direction of the connectivity effect between the amygdala and the mPFC (and other regions). Recently, however, a few studies have analyzed the directed functional connectivity, i.e., effective connectivity, of the amygdala in PTSD patients. Gilboa et al. utilized a structural equation model (SEM) to study the effective connectivity of the amygdala and found that, in PTSD patients, the mPFC did not significantly alter the patients' amygdala, but the influence of the amygdala on the mPFC and the visual cortex was enhanced (18). The Gilboa study had its limitations: the task was designed in a symptom provocation-dependent manner and the SEM required prior hypotheses. In addition, some PTSD patients had a history of drug treatment during the study.

We aimed to use resting-state fMRI and Granger causality analysis to observe the change in the effective connectivity of the amygdala in PTSD patients, and to correlate that with the severity of PTSD symptoms. Compared to other modeling methods for effective connectivity (SEM or a dynamic causality model, for example), the Granger causality analysis does not require researchers to pre-select the interaction areas, and this advantage makes it popular for analyzing the effective connectivity of many diseases, including depression, mild cognitive impairment, and chronic tinnitus, among others (19–21). Recent studies have also reported that trauma could cause changes in brain area functions, as well as in functional connectivity between different areas (22, 23); thus, our study also included healthy volunteers who had not experienced trauma, in order to analyze the influence of trauma on the effective connectivity of the amygdala.

## Methods

### Participants and clinical assessment

On July 18, 2014, Typhoon Rammasun, a category 5 super typhoon struck Wenchang city on the island province of China. People residing in this area were heavily affected by this typhoon, which caused at least 14 deaths. Particularly, in Luodou farm of Wenchang city, more than 1000 people were trapped and almost drowned by the storm tide induced by this destructive typhoon. We recruited 70 typhoon-exposed subjects from this area, 36 with PTSD (nine males and 27 females) and 34 without PTSD (trauma exposed controls [TEC], seven males and 27 females), who were all screened with the PTSD Checklist-Civilian Version (PCL-C) (24). Two trained and reliable psychiatric specialists conducted all the neuropsychiatric investigations. The PTSD diagnosis was based on DSM-IV diagnostic criteria for current PTSD, and symptoms were assessed with the Clinician-Administered PTSD Scale (CAPS) (25). The CAPS for DSM-IV is a structured interview that assesses the frequency and intensity of each PTSD symptom using a behaviorally anchored rating (from 0 to 4). This scale assesses the 17 core PTSD symptoms listed in the DSM-IV and obtains information regarding symptom onset, duration, and functional impact. The absence or presence of comorbid disorders was determined via the Structural Clinical Interview for DSM-IV. Furthermore, 32 healthy volunteers (HC, nine males and 23 females) who did not meet DSM-IV Criterion A1 for PTSD were recruited via advertisement from Haikou, a city about 35 km from Wenchang city. For all participants, the Self-Rating Anxiety Scale (SAS) (26) and Self-Rating Depression Scale (SDS) (27) were administered to assess anxiety and depression symptoms, respectively. All the above procedures occurred between November 2014 and January 2015.

General exclusion criteria included age <18 years or >65 years, left-handedness, a history of head injury or loss of consciousness, significant medical and neurological conditions, comorbid lifetime or current psychiatric disorders other than depression and anxiety, alcohol or drug abuse/dependence, use of anti-depressants or any form of psychotherapy, and contraindications to MRI, such as claustrophobia, pregnancy, and ferromagnetic implants. In the PTSD group, completed imaging data were not available for three female subjects, and six were removed for denture-related artifacts (one female, one male), brain infarction revealed by conventional MRI (one female), pregnancy (one female), and excessive movement during MRI scanning (translation >1.5 mm or rotation >1.5° in any direction, one male and one female). In addition, we excluded one female TEC for excessive movement and two male HCs for brain infarction. Thus, 27 PTSD patients, 33 TECs, and 30 HCs were ultimately included in the statistical analysis. The study was conducted in accordance with the declaration of Helsinki and was approved by the ethics committee of Hainan General Hospital and the Second Xiangya Hospital of Central South University. All participants provided written, informed consent after a detailed description of this study.

### MRI data acquisition

A 3.0 Tesla whole-body MRI scanner (Magnetom Tim Skyra, Siemens Medical Solutions, Erlangen, Germany) with a 32-channel phased array head coil was used for image acquisition. Subjects' heads were immobilized using a foam pad and a Plexiglas head cradle. High-resolution, T1-weighted, 3D anatomical images were also acquired with a sagittal magnetization-prepared rapid gradient echo sequence for later co-registration and normalization (TR/TE = 2300/1.97 ms, flip angle = 9°, FOV = 256 × 256 mm, matrix = 256 × 256, 176 slices, slice thickness = 1 mm, the total time points = 353 s). BOLD fMRI was prescribed parallel to the anterior commissure-posterior commissure line, which was acquired using a gradient-echo planar imaging (EPI) sequence with an interleaved slice excitation order and a 2 mm isotropic spatial resolution (FOV = 230 × 230 mm, matrix = 64 × 64, TR/TE = 2,000 ms/30 ms, flip angle = 90°, 35 slices, slice thickness = 3.6 mm, no intersection gap, total volume number = 250, the total time points = 508 s). During the functional scanning, subjects were instructed to lie quietly, keep their eyes closed, and let their mind wander without falling asleep.

### Data preprocessing

The imaging data were preprocessed using Statistical Parametric Mapping software (SPM8, http://www.fil.ion.ucl.ac.uk/spm/). The first 10 volumes of the functional images were discarded to ensure signal equilibrium. The remaining 240 volumes were slice-time-corrected, realigned, and co-registered with the anatomical scan. The co-registered anatomical images were then segmented into gray matter (GM), white matter (WM), and cerebrospinal fluid (CSF), and normalized into standard Montreal Neurological Institute (MNI) space, with a final size of 3 × 3 × 3 mm^3^ (28). The resulting normalization matrix was then applied to the functional data. After that, the functional images were smoothed by convolution with an isotropic Gaussian kernel (full width at half maximum [FWHW] = 8 mm). After smoothing, the imaging data were filtered (bandpass, 0.01–0.08 Hz) to remove the effects of low-frequency drift and high-frequency noise. Finally, nuisance covariates, including cerebrospinal fluid signals, global mean signals, white matter signals, and head motion parameters, were regressed out from the fMRI data.

### Effective connectivity analysis

In this study, the SPM8 Anatomy toolbox was used to select the bilateral amygdala (two parts, including the basolateral amygdala and central medial amygdala) as a region of interest (ROI) using 3 × 3 × 3 mm^3^ resampling normalization. The bilateral amygdala was set as a seed region using the WFU_PickAtlas software (http://www.ansir.wfubmc.edu). Effective connectivity was analyzed using REST-GCA in the REST toolbox (29). In this study, the time series for the bilateral amygdala was defined as the seed time series x, and the time series y denoted the time series of all voxels in the brain. The linear direct influence of x on y (Fx → y), and the linear direct influence of y on x (Fy → x) were calculated voxel-by-voxel across the brain. Thus, two Granger causality maps were generated based on the influence measures for each subject. The residual-based F was normalized (F′) and standardized to a Z score for each voxel (Zx → y and Zy → x, subtracting the global mean F' values, divided by the standard deviation).

### Statistical analysis

The chi-squared test was used to analyze gender distribution, and a one-way analysis of variance (ANOVA) was performed for all continuous variables except for PCL scores, for which an independent *t*-test was used to examine differences between the PTSD group and the TEC group. The above analyses were conducted with SPSS version 16.0 (SPSS Inc., Chicago, IL, USA), with the significance threshold set at *P* < 0.05.

We used SPM8 to analyze the GCA maps of the three groups. Within each group, a random effect, one-sample *t*-test was used (*P* < 0.05, AlphaSim-corrected). The effective connectivity differences between the amygdala and the whole brain among the three groups were analyzed using an ANOVA with education level and depression diagnosis as covariates, followed by *post hoc t*-tests to examine the between-group differences if a statistical difference was noted (*P* < 0.05, AlphaSim-corrected).

To investigate the association between PTSD symptom severity and brain measures, mean GCA (*Z* values) from clusters with significant group differences were extracted from *post hoc t*-tests, and then correlated against the CAPS total scores using Pearson correlation analysis. The correlation analysis was accomplished with SPSS, with a significant threshold of *P* < 0.05 (not corrected).

## Results

### Demographic and clinical variables

The demographic and clinical characteristics are summarized in Table 1. There was no significant difference in age (*F* = 0.317, *P* = 0.729) or gender distribution (*P* = 0.912) among the PTSD, TEC, and HC groups. The education level of the HC group was higher than that of the PTSD group and the TEC group (*F* = 8.396, *P* < 0.001). The time from the typhoon to the examination was 105.5 ± 9.5, 118.0 ± 10.0, and 125.8 ± 1.0 days, respectively, in the PTSD, TEC, and HC groups. The mean CAPS total score of the PTSD group was 78.2 ± 19.3, and the PCL scores were higher in the PTSD group compared to the TEC group (*P* < 0.001). Ten PTSD patients had current psychiatric co-morbidity: nine with depression (two males and seven females) and one with generalized anxiety disorder (one female). Significant differences were also found among the three groups in the SAS (*F* = 81.864, *P* < 0.001) and SDS scores (*F* = 101.915, *P* < 0.001). *Post hoc* analyses revealed that the SAS and SDS scores in the TEC group were significantly higher than those in the HC group, but were significantly lower compared to those of the PTSD group.

**Table 1:** Demographic and clinical data of traumatized individuals and healthy controls.

	**PTSD (*n* = 27)**	**TEC (*n* = 33)**	**HC (*n* = 30)**	***P*-value**
Gender (males/females)	7/20	7/26	7/23	0.912^a^
Age (year)	48.4 ± 10.3	48.5 ± 7.5	49.9 ± 6.1	0.729^b^
Education (year)	6.4 ± 3.4	7.0 ± 3.4	9.7 ± 3.3	< 0.001^b^
Days after the disaster to exam	105.5 ± 9.5	118.0 ± 10.0	125.8 ± 1.0	< 0.001^b^
SAS score	65.8 ± 13.3	41.3 ± 8.1	36.0 ± 5.5	< 0.001^b^
SDS score	69.6 ± 13.2	41.3 ± 9.1	33.5 ± 7.2	< 0.001^b^
PCL score	53.7 ± 8.5	28.9 ± 5.4		< 0.001^c^
CAPS total score	78.2 ± 19.3			

a *P-value obtained with chi-square test*.

b *P-value obtained with one-way analysis of variance*.

c *P-value obtained with independent t-test for continuous variables. Values are given as mean ± SD except for gender, which is presented as a number. PTSD, post-traumatic stress disorder; TEC, trauma-exposed control; HC, healthy control; SAS, Self-Rating Anxiety Scale; SDS, Self-Rating Depression Scale; PCL, PTSD Checklist; CAPS, Clinician-Administered PTSD Scale*.

#### Effective connectivity from the left amygdala

**Figure 1:**
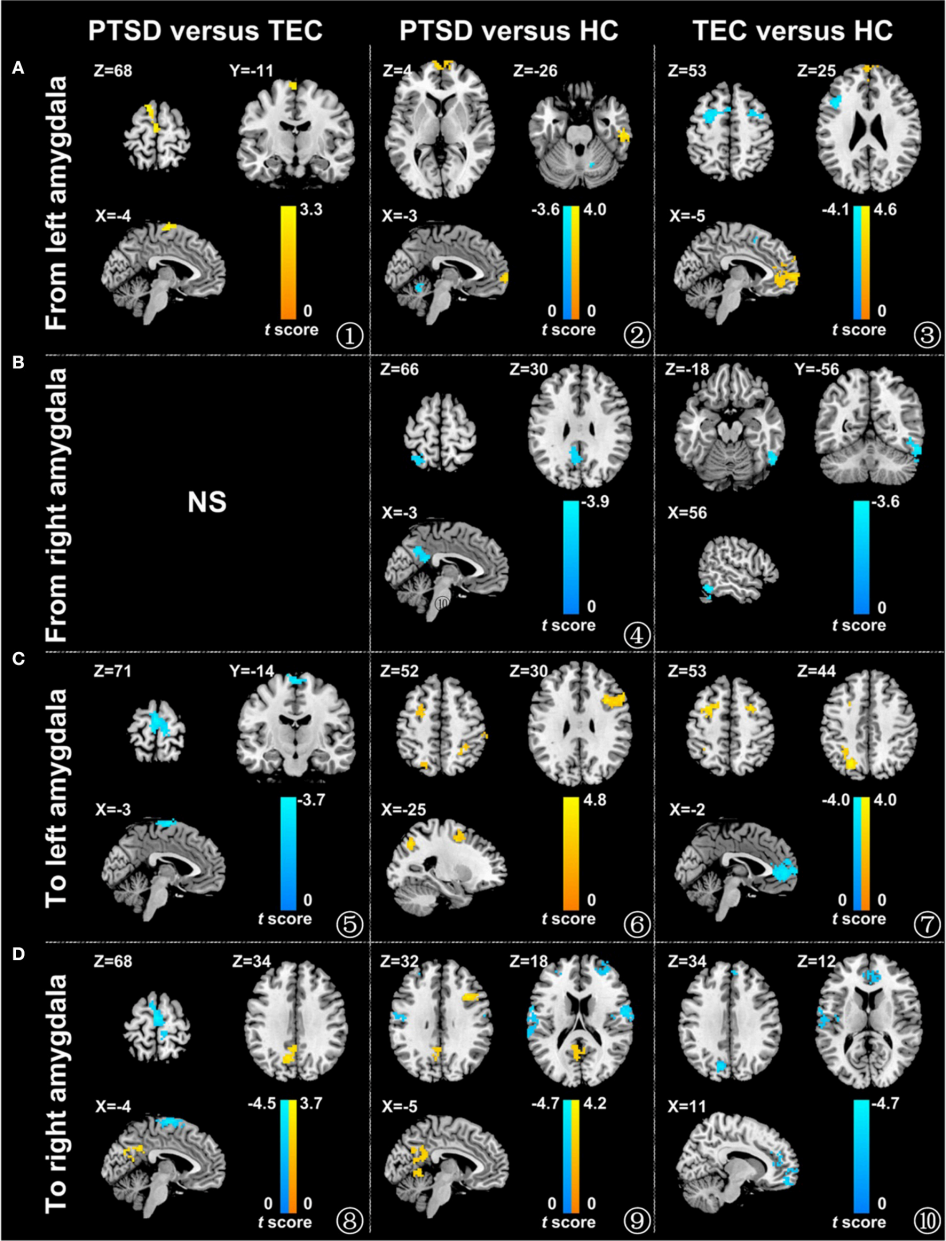
Comparison of the effective connectivity between the amygdala and different brain areas in the different groups. The influence of the left amygdala on the whole brain **(A)**, the influence of the right amygdala on the whole brain **(B)**, the influence of the whole brain on the left amygdala **(C)**, and the influence of the whole brain on the right amygdala **(D)** (*P* < 0.05, adjusted by AlphaSim). The warm color represents the positive functional connectivity; the cold color represents the negative functional connectivity. PTSD, post-traumatic stress disorder group; TEC, trauma-exposed control group; HC, healthy control group.

**Figure 2:**
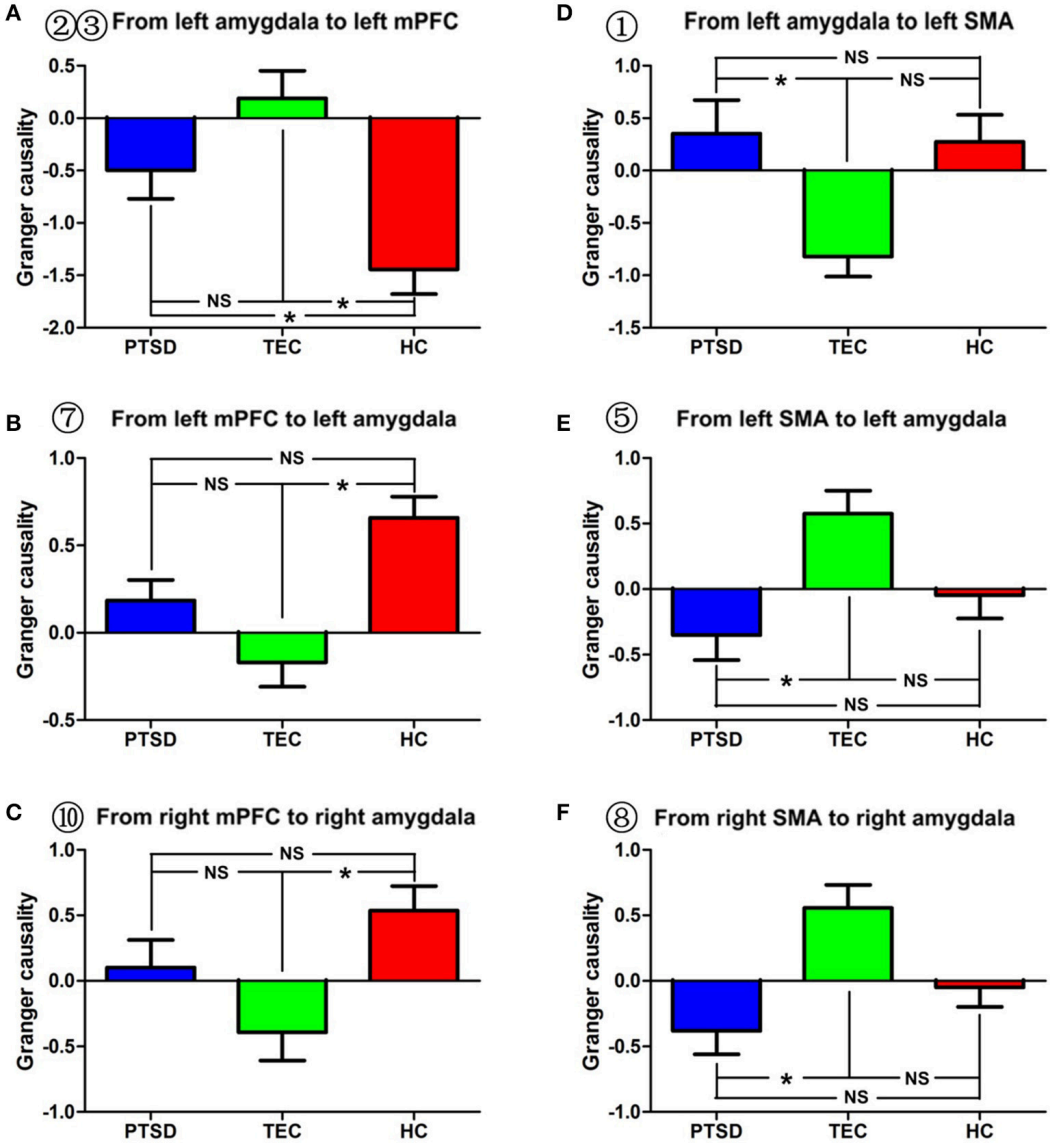
Effective connectivity between the amygdala and between the amygdala and the mPFC and between the amygdala and the SMA in the different groups. **(A–C)** indicate inter-group differences regarding the influence of the amygdala on the mPFC and the influence of the mPFC on the amygdala. **(D–F)** indicate inter-group differences regarding the influence of the amygdala on the SMA and the influence of the SMA on the amygdala. PTSD, post-traumatic stress disorder group; TEC, trauma-exposed control group; HC, healthy control group; vmPFC, ventral media prefrontal cortex; SMA, supplementary motor area. “^*^” means having significant difference between the two groups.

**Figure 3:**
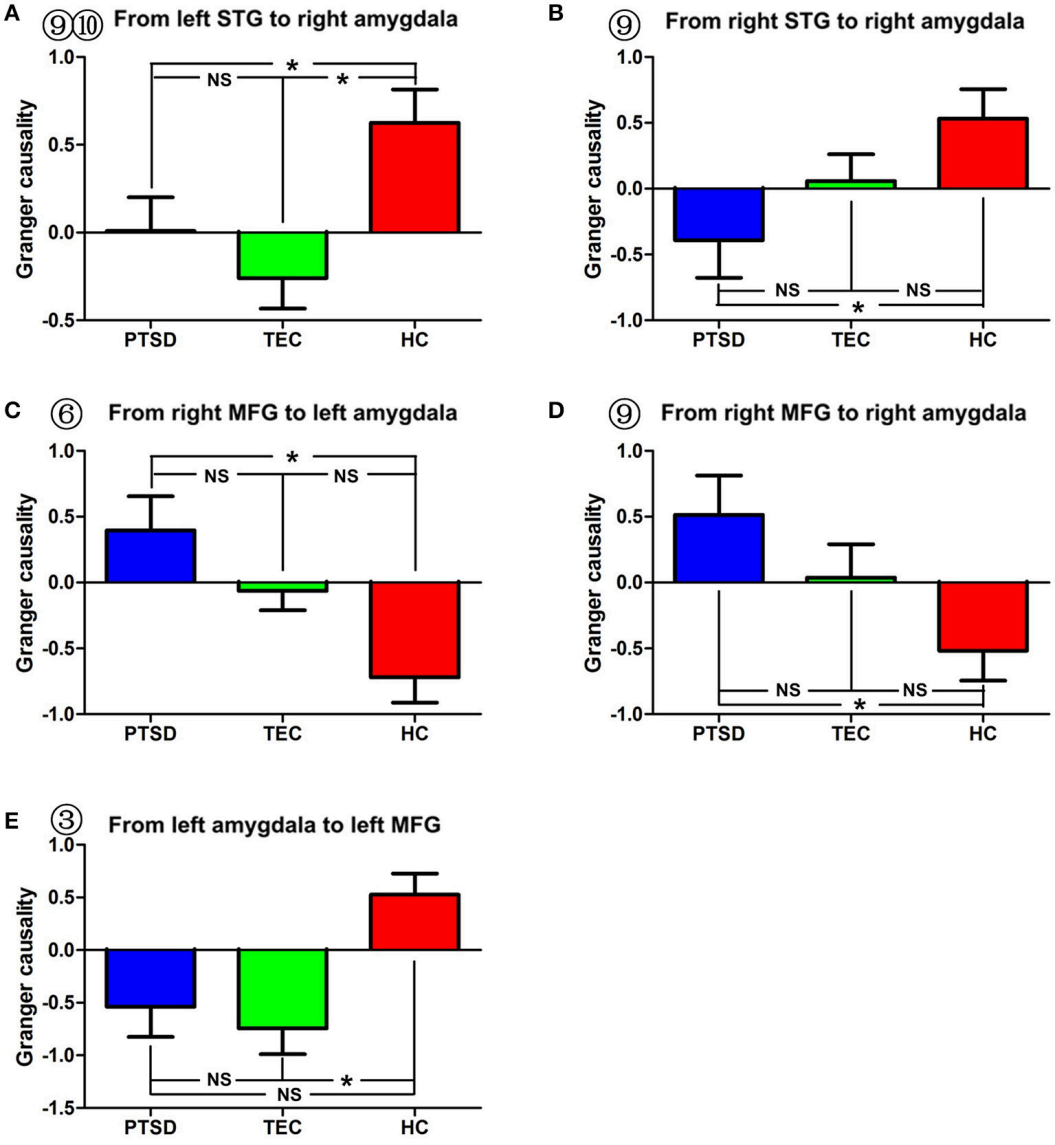
Comparison of effective connectivity between the amygdala and the bilateral superior temporal gyrus and between the amygdala and the middle temporal gyrus. **(A,B)** show inter-group differences regarding the influence of the superior temporal gyrus on the right amygdala. **(C–E)** show inter-group differences regarding the influence of the middle frontal gyrus on the amygdala and the influence of the amygdala on the middle frontal gyrus. PTSD, post-traumatic stress disorder group; TEC, trauma-exposed control group; HC, healthy control group; SFG, superior frontal gyrus; MFG, middle frontal gyrus. “^*^” means having significant difference between the two groups.

**Figure 4:**
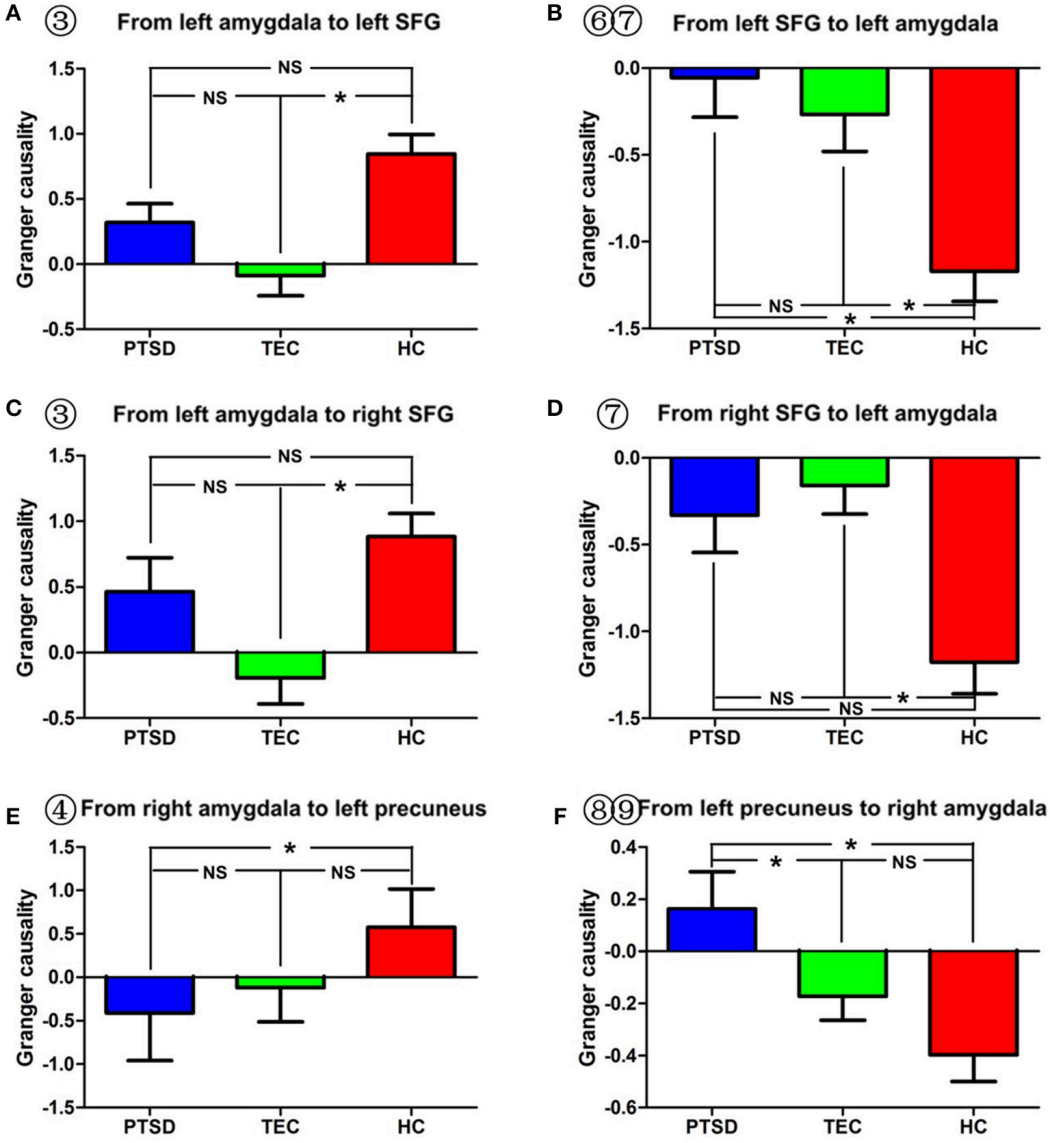
Comparison of effective connectivity between the amygdala and the superior temporal gyrus and between the amygdala and the precuneus. **(A–D)** Show inter-group differences regarding the influence of the amygdala on the superior temporal gyrus and the influence of the superior temporal gyrus on the amygdala. **(E,F)** Indicate inter-group differences regarding the influence of the amygdala on the precuneus, and the influence of the precuneus on the amygdala. PTSD, post-traumatic stress disorder group; TEC, trauma-exposed control group; HC, healthy control group; SFG, superior frontal gyrus. “^*^” means having significant difference between the two groups.

A significant difference in effective connectivity between the left amygdala and the left supplementary motor area (SMA) was observed in the PTSD vs. the TEC group (Table 2, Figure 1A). Although a positive causal effect of the left amygdala on the left SMA was observed in the PTSD group, a negative causal effect was observed in the TEC group (Figure 2D). Significant differences in effective connectivity between the left amygdala and the bilateral ventral medial prefrontal cortex (vmPFC), and between the left amygdala and the right inferior temporal gyrus (ITG) were observed in the PTSD vs. the HC group (Table 2, Figure 1A). A negative causal effect of the left amygdala on the left mPFC was observed in both the PTSD group and the HC group (Figure 2A). Significant differences in effective connectivity between the left amygdala and many brain regions, including the vmPFC, the left superior frontal gyrus (SFG), the right SFG, and the left middle frontal gyrus (MFG), were observed in the TEC vs. the HC group (Table 2, Figure 1A). The positive causal effect of the left amygdala on the mPFC, and the negative causal effect of the left amygdala on the left SFG, the right SFG, and the left MFG were observed in the TEC group. The positive causal effect of the left amygdala on the left SFG, the right SFG, the left MFG, as well as the negative causal effect of the left amygdala on the mPFC, were observed in the HC group (Figures 2A,3E,4A,C).

**Table 2:** Comparison of effective connectivity from the amygdala.

**Brain regions**	**MNI coordinates (mm) (x, y, z)**	**Voxel number**	***t*-value**
**PTSD–TEC**			
Left SMA/paracentral lobule	−3, −9, 72	66	3.17
**PTSD–HC**			
Bilateral vmPFC	−3, 69, 3	79	3.16
Right ITG/MTG	63, −24, −24	99	4.04
**TEC**–**HC**			
Bilateral vmPFC	−3, 66, 3	448	4.57
Left SFG	−18, 6, 51	149	−3.99
Right SFG	42, 0, 60	97	−3.96
Left MFG	−42, 15, 24	65	−4.13

*PTSD, post-traumatic stress disorder group; TEC, trauma-exposed control group; HC, healthy control group; MNI, Montreal Neurologic Institute; SMA, supplementary motor area; vmPFC, ventral medial prefrontal cortex; ITG, inferior temporal gyrus; MTG, middle temporal gyrus; SFG, superior frontal gyrus; MFG, middle frontal gyrus*.

#### Effective connectivity from the right amygdala

As for the effective connectivity from the right amygdala, no significant difference was identified between the PTSD and the TEC groups. However, significant differences in effective connectivity between the right amygdala and the left precuneus/posterior cingulate gyrus (PCC) and between the right amygdala and the superior parietal lobule (SPL) were observed in the PTSD group vs. the HC group (Table 3, Figure 1B). There was also a significant difference in effective connectivity between the right amygdala and the inferior temporal gyrus (ITG) in the TEC group vs. the HC group (Table 3, Figure 1B). There was a positive causal effect of the right amygdala on the left precuneus/PCC in the HC group, while negative causal effects were observed in these two areas in the PTSD and TEC groups (Figure 4E).

**Table 3:** Comparison of the effective connectivity from the right amygdala.

**Brain regions**	**MNI coordinates (mm) (x, y, z)**	**Voxel number**	***t* value**
**PTSD–HC**			
Left precuneus/PCC	−6, −48, 21	116	−3.64
Left SPL	−24, −51, 72	73	−3.87
**TEC**–**HC**			
Right ITG	57, −57, −18	68	−3.60

*PTSD, post-traumatic stress disorder group; MNI, Montreal Neurologic Institute; ITG, inferior temporal gyrus; PCC, posterior cingulate cortex; SPL, superior parietal lobule*.

#### Effective connectivity to the left amygdala

A significant difference in effective connectivity between the bilateral SMA and the left amygdala was observed in the PTSD group vs. the TEC group (Table 4, Figure 1C). While there was a positive causal effect of the left SMA on the left amygdala in the TEC group, negative causal effects were observed in the PTSD and HC groups (Figure 2E). There were significant differences in effective connectivity between the left SFG and the left amygdala, the right MFG and the left amygdala, and the bilateral SPL and the left amygdala in the PTSD group vs. the HC group (Table 4, Figure 1C). Significant differences in effective connectivity between the bilateral SFG and the left amygdala, the mPFC and the left amygdala, and the left SPL and the left amygdala were also observed in the TEC group vs. the HC group (Table 4, Figure 1C). There were also positive causal effects of the left mPFC on the left amygdala in the PTSD group and the HC group, as well as for the right MFG on the left amygdala in the PTSD group. There were negative causal effects of the left mPFC on the left amygdala in the TEC group, and for the right mPFC on the left amygdala in the TEC group and the HC groups, as well as for the bilateral SFG on the left amygdala in all three groups (Figures 2B,3C,4B,D).

**Table 4:** Comparison of the effective connectivity to the left amygdala.

**Brain regions**	**MNI coordinates (mm) (x, y, z)**	**Voxel number**	***t* value**
**PTSD–TEC**			
Bilateral SMA/paracentral lobule	−6, −24, 72	133	−3.73
**PTSD–HC**			
Left SPL/MOG	−24, −69, 42	76	4.01
Right SPL/IPL	33, −48, 39	82	3.69
Left SFG	−24, −3, 48	66	3.90
Right MFG	36, 15, 27	165	4.77
**TEC**–**HC**			
Bilateral vmPFC	−9, 51, 0	526	−4.02
Left SFG	−18, 9, 48	131	3.77
Right SFG	27, 3, 60	65	3.64
Left SPL/MOG	−24, −66, 45	89	4.01

*PTSD, post-traumatic stress disorder group; TEC, trauma-exposed control group; HC, healthy control group; MNI, Montreal Neurologic Institute; SMA, supplementary motor area; SPL, superior parietal lobule; MOG, middle occipital gyrus; IPL, inferior parietal lobule; SFG, superior frontal gyrus; MFG, middle frontal gyrus; vmPFC, ventral medial prefrontal cortex*.

#### Effective connectivity to the right amygdala

There were significant differences in effective connectivity between the bilateral SMA and the right amygdala, between the left precuneus/PCC and the right amygdala in the PTSD group vs. the TEC group (Table 5, Figure 1D). There were positive causal effects of the left SMA on the right amygdala in the TEC group and negative causal effects of the left SMA on the right amygdala in the PTSD and HC groups (Figure 2F).

**Table 5:** Comparison of effective connectivity to the right amygdala.

**Brain regions**	**MNI coordinates (mm) (x, y, z)**	**Voxel number**	***t* value**
**PTSD**–**TEC**			
Left precuneus/PCC	−3, −51, 33	88	3.15
Bilateral SMA/paracentral lobule	3, −12, 69	179	−3.94
**PTSD–HC**			
Left precuneus/PCC	−3, −51, 21	85	3.32
Right MFG	39, 12, 27	70	4.16
Left STG/postcentral gyrus	−57, −21, 6	176	−3.44
Right STG/postcentral gyrus	63, −6, 15	156	−4.17
Left MFG	−30, 39, 24	62	−3.37
Right MFG	30, 51, 3	70	−3.69
**TEC–HC**			
Bilateral vmPFC	12, 54, −15	76	−3.82
Left STG/postcentral gyrus	−57, −12, 15	110	−3.75
Left OFC	−27, 42, −15	93	−3.85
Right OFC	18, 54, −12	63	−4.73
Left cuneus/precuneus	−9, −75, 33	77	−3.87

*PTSD, post-traumatic stress disorder group; TEC, trauma-exposed control group; HC, healthy control group; MNI, Montreal Neurologic Institute; PCC, posterior cingulate cortex; SMA, supplementary motor area; MFG, middle frontal gyrus; STG, superior temporal gyrus; MFG, middle frontal gyrus; vmPFC, ventral medial prefrontal cortex; SPL, superior parietal lobule; MOG, middle occipital gyrus; IPL, inferior parietal lobule; SFG, superior frontal gyrus; vmPFC, ventral medial prefrontal cortex; OFC, orbital frontal cortex*.

Significant differences in effective connectivity between the left precuneus/PCC and the right amygdala, between the bilateral MFG and the amygdala, and between the bilateral STG and the right amygdala were observed in the PTSD group vs. the HC group (Table 5, Figure 1D). There were also significant differences in effective connectivity between the bilateral vmPFC and the right amygdala, between the bilateral OFG and the right amygdala, between the left STG/postcentral gyrus and the right amygdala, and between the left cuneus/precuneus and the right amygdala in the TEC group vs. the HC group (Table 5, Figure 1D). There were positive causal effects of the right mPFC on the right amygdala in the PTSD group and in the HC group, and for the right STG on the right amygdala in the TEC group and in the HC group. There were positive causal effects also for the left STG on the right amygdala in the PTSD group and in the HC group, for the right MFG on the right amygdala in the PTSD group and in the TEC group, and for the left precuneus/PCC on the right amygdala in the PTSD group. There were negative causal effects of the right mPFC on the right amygdala in the TEC group, as well as for the right STG on the right amygdala in the PTSD group, the left STG on the right amygdala in the TEC group, the right MFG on the right amygdala in the HC group, and for the left precuneus/PCC on the right amygdala in both the TEC and HC groups (Figures 2C,3A,B,D,4F).

#### Correlation analysis

Pearson correlation analysis showed that the strength of effective connectivity (average *Z* value) in the brain areas that demonstrated inter-group differences in effective connectivity between the amygdala and other brain regions showed no significant correlation with the total CAPS score.

## Discussion

Based on previous functional connectivity research, this study used fMRI and Granger causality analysis to compare the effective connectivity of the amygdala between healthy volunteers and those who had experienced a typhoon. The results showed that PTSD patients and TECs had altered effective connectivity between the amygdala and the mPFC, between the amygdala and the SMA, between the amygdala and the dlPFC, between the amygdala and the STG, and between the amygdala and the precuneus/PCC, indicating that PTSD and trauma may cause abnormal integration of functions between the amygdala and other multiple brain regions.

In recent years, many studies have reported altered functional connectivity between the amygdala and the mPFC in patients with PTSD. Researchers believe that this may reflect abnormal regulation of the amygdala by the mPFC in PTSD (15, 16, 30). However, in our study, we identified an increased inhibition of the amygdala by the mPFC only in the TEC group (compared to the healthy controls). The mPFC inhibited amygdala activity in the TEC group, whereas the mPFC promoted amygdala activity in the healthy control group. Although, in PTSD patients, the mPFC also exerted an activation effect on the amygdala, no significant difference with regard to the influence of the mPFC on the amygdala was identified between the PTSD and the TEC groups. This suggests that, in the TEC group, the mPFC exerted greater inhibition on the amygdala, which might be a protective factor for PTSD, and this partially explains the reason that, despite experiencing the typhoon, this group did not develop PTSD. Grant et al. performed conditioned and unconditioned auditory stimuli task-based fMRI to analyze the influence of trauma on the effective connectivity of the amygdala, and found that individuals who have experienced childhood trauma showed basal lateral amygdala inhibition (BLA) by the mPFC, whereas healthy controls did not show such inhibition, indicating that increased inhibition of the amygdala by the mPFC is a manifestation of an active response to traumatic stress (31).

Interestingly, when compared to the healthy controls, PTSD patients and the TECs in our study showed decreased inhibition of the mPFC by the amygdala or even activation of the mPFC. This indicates that trauma might cause an enhanced ascending drive of the mPFC by the amygdala. Stein et al. analyzed the effective connectivity of the amygdala in healthy volunteers using a fearful face fMRI paradigm and found that the amygdala activated the mPFC in a bottom-up manner (32). Gilboa et al. also reported that the amygdala could promote the activity of the mPFC, and the ascending excitation of the mPFC by the amygdala in PTSD patients was significantly stronger than that in the TEC group (18). Our results showed no significant difference between the PTSD and TEC groups in terms of the influence of the amygdala on the mPFC. This could be explained by different imaging techniques, experimental paradigms, as well as subject selection. Our study used resting-state fMRI and selected subjects who had experienced the same trauma, but, in the Gilboa et al. study, they used PET and symptom-provoked tasks, and the subjects experienced different traumatic events. In fact, some of the PTSD patients took clonazepam during the test (18). Pezawas et al. believed that, under fear tasks (e.g., the fearful face fMRI paradigm), there is a negative feedback circuit of the amygdala in healthy volunteers; namely, the amygdala transfers related emotional information to the mPFC in an ascending manner, and the mPFC inhibits the amygdala in a descending manner (33). Thus, our study analyzed the bidirectional influence between the amygdala and the mPFC and found that healthy volunteers also display such a feedback loop under resting-state conditions, which, however, is different from the negative feedback loop during the task shown in Pezawas's study. As demonstrated in our study, the mPFC is inhibited by the amygdala in healthy controls, while trauma seems not only to reduce such inhibition, but also to cause the amygdala to activate the mPFC. Meanwhile, the amygdala is activated by the mPFC in healthy controls, while trauma could reduce this activation and even turn it into inhibition of the amygdala by the mPFC. As a result, the relative insufficient activation of the mPFC by the amygdala and insufficient inhibition of the amygdala by the mPFC might lead to PTSD in those who have been exposed to traumatic incidents. Anatomically, the uncinate fasciculus is the major white matter fasciculus that connects the amygdala and the mPFC. Based on diffusion tensor imaging, Costanzo et al. analyzed the brain structure of sub-clinical PTSD patients and identified a negative correlation between the PTSD symptom score and the integrity of the uncinate fasciculus (34). This suggests that changes in effective connectivity between the amygdala and the mPFC may have a structural basis. Our study also found that, when compared to healthy controls, the activation of the dlPFC by the amygdala became inhibition in the TEC group, and the inhibition of the amygdala by the dlPFC was significantly decreased in both the PTSD and the TEC groups. The PTSD group had less inhibition of the amygdala by the dlPFC when compared with the TECs, but no significant difference was identified between the two groups. In addition, the PTSD group showed greater activation of the SMA by the amygdala and greater inhibition of the amygdala by the SMA compared to the TEC group. Partially consistent with our results, structural MRI studies have revealed significantly decreased gray matter volume in the SMA of PTSD patients compared to healthy controls (35). MacNamara et al. used fMRI and an emotional regulation task in a PTSD follow-up study of veterans who were undergoing serotonin reuptake inhibitors (SSRIs) treatment, and found that PTSD patients had significantly increased SMA activation after being treated with a 5-hydroxytryptamine reuptake inhibitor (36). Many neuroimaging studies revealed that, either with or without PTSD, all individuals exposed to trauma had an abnormal dlPFC structure (gray matter volume, cortex thickness), an abnormal regional brain function, or altered functional connectivity (7, 10, 12, 37). In our study, we also found that PTSD patients showed decreased negative effective connectivity between the amygdala and the middle frontal gyrus (MFG) compared to controls. The dlPFC and the SMA are responsible for implementation, attention, and working memory (38)—the dlPFC plays an important role in exciting and maintaining emotion regulation (39), and the SMA participates in motor control and mediates emotion regulation by the former. Thus, our study showed that trauma could cause a change in interaction between the dlPFC and the amygdala that ultimately results in lessened inhibition of the latter by the former. However, based on the results of previous PTSD studies, we believe that PTSD might aggravate the influence of trauma on emotional regulation by the dlPFC. It should be noted that, in animal experiments and diffusion tensor imaging of healthy volunteers, there was no direct white matter connection between the amygdala and the dlPFC, which indicates the effective connectivity between the two may be indirect rather than direct (40). Nevertheless, in our study, the change in effective connectivity between the amygdala and the SMA in PTSD patients suggested increased regulation by the SMA of the amygdala, compared to the results in the TEC group. Since PTSD patients demonstrated insufficient inhibition of the amygdala by the mPFC and dlPFC, we speculate that the increased regulation of the amygdala by the SMA in the PTSD group could be a compensation for the insufficient inhibition of the amygdala by the mPFC and dlPFC.

In addition, our study found that the PTSD and the TEC groups showed greater inhibition of the amygdala by the STG compared to that in the healthy controls. These results indicate that abnormal functional and effective connectivity between the amygdala and the STG is a trauma-associated brain function change. Structural MRI studies of PTSD found that, compared to healthy controls, patients exposed to trauma had increased gray matter volume or decreased cortical thickness in their STG (41, 42). Zhou et al. followed car accident survivors using resting-state fMRI and reported that the functional connectivity between the STG and the PCC was negatively associated with the severity of PTSD several months after the accident (43). Another resting-state MRI study found that subjects who had experienced childhood trauma demonstrated a decreased regional homogeneity of their STG compared to healthy controls (44). A novel method with which to evaluate resting-state activity is through the estimation of regional homogeneity, which provides an estimation of the efficiency of coordinated neuronal activity. From these conclusions, it appears that previous PTSD or trauma-related studies have reported results similar to those of our study, and that trauma does cause structural and functional changes of the STG. The STG is associated with hearing, semantic processing, and episodic memory (41), and is responsible for regulating the activity of the amygdala, thereby playing a significant role in reducing conditioned fear (45). Structural studies have shown that a wide range of bidirectional projection fibers are present between the STG and the limbic system, such as between the STG and the hippocampus, and the STG and the amygdala (41); thus, we believe that, in our study, increased amygdala inhibition by the STG in those who have experienced traumatic events also reflected a compensatory mechanism for the regulation of emotion by the human brain.

The precuneus/PCC are key brain areas for the default network, and they play important roles in visual spatial imagination, self-referential processing, and autobiographical memory (46). In this study, we found that, when compared with the TECs and the healthy controls, PTSD patients showed decreased inhibition of the amygdala by the precuneus/PCC; when compared with the healthy controls, the PTSD group showed increased inhibition of the precuneus/PCC by the amygdala. Stein et al. used a fearful face fMRI paradigm to assess the effective connectivity of the amygdala in healthy volunteers, and found an inhibitive effect of the precuneus/PCC on the amygdala (32). The precuneus and surrounding posteromedial areas are involved in the interwoven network of the neural correlates of self-consciousness, and are engaged in self-centered mental imagery strategies and episodic memory retrieval (47). Young et al. used an autobiographical memory task to examine the functional connectivity of the amygdala in depressed and vulnerable individuals, and found that depressed participants exhibited significantly decreased amygdala connectivity with the precuneus/PCC during positive recall. Thus, we speculate that the decreased inhibition of the amygdala by the precuneus/PCC could be associated with abnormal regulation of fear, whereas the increased inhibition of the precuneus/PCC by the amygdala may be associated with the impairment of autobiographical memory. Partially consistent with our results, many resting-state fMRI studies also identified abnormal functional connectivity between the amygdala and the precuneus/PCC in PTSD patients (15, 30, 48). Also, since the amygdala is an important node for the salience network, abnormal effective connectivity between the amygdala and the precuneus/PCC, as identified in that study, also indicates the loss of balance between the salience and default networks and might be associated with abnormal emotion regulation and impaired autobiographical memory in PTSD patients (7, 49).

This study also has some limitations. First, we chose only the bilateral amygdala as the ROI and did not analyze the effective connectivity of the sub-areas of the amygdala; thus, future work should further explore the changes in effective connectivity in the sub-areas of the amygdala. Second, the low time resolution of fMRI and the delayed hemodynamic response may have affected the results of the Granger causality analysis. Since the Granger causality is not equivalent to the interaction between neurons, the combination of brain structural studies at either the cellular or molecular levels would help to further clarify the neural mechanism of PTSD. Finally, although the simultaneous inclusion of trauma and healthy controls helped to elucidate whether changes in brain functions were PTSD- or trauma-associated, the cross-sectional study design made it difficult for us to distinguish whether PTSD-related abnormal effective connectivity of the amygdala is a risk factor for disease or an acquired change.

In conclusion, based on the Granger causality analysis, this study found that both PTSD and trauma caused changes in effective connectivity between the amygdala and many brain regions, including the mPFC, the dlPFC, the SMA, the STG, and the precuneus/PCC. Trauma could lead to increased ascending activation of the mPFC by the amygdala, and decreased regulation of the amygdala by the dlPFC. The greater inhibition of the amygdala by the mPFC may serve as a protective factor for PTSD, and altered amygdala-SMA and amygdala-STG effective connectivity may reflect compensatory mechanisms of brain function. These data raise the possibility that insufficient inhibition of the amygdala by the mPFC might lead to PTSD in those who have been exposed to traumatic incidents, and may inform future therapeutic interventions.

## Author contributions

FC and JK collected the data, performed the analysis and wrote the manuscript. RQ contributed to the design of the study. TL collected the MRI data. QX contributed to the design of the study. YZ contributed to the discussion and manuscript revision. JL revised the manuscript for intellectual content. GL and LZ is the guarantor of this work and, as such, had full access to all the data in the study and takes responsibility for the integrity of the data and the accuracy of the data analysis. All authors reviewed the manuscript.

### Conflict of interest statement

The authors declare that the research was conducted in the absence of any commercial or financial relationships that could be construed as a potential conflict of interest.
